# The influence of nutrient enrichment on riverine food web function and stability

**DOI:** 10.1002/ece3.7107

**Published:** 2020-12-21

**Authors:** Adam D. Canning, Russell G. Death

**Affiliations:** ^1^ Centre for Tropical Water and Aquatic Ecosystem Research (TropWATER) James Cook University Townsville Qld Australia; ^2^ School of Agriculture and the Environment Massey University Palmerston North New Zealand

**Keywords:** ecological network, energy flow, food web, indirect effects, mutualism, nutrients, productivity, respiration, river, stability

## Abstract

Nutrient enrichment of rivers and lakes has been increasing rapidly over the past few decades, primarily because of agricultural intensification. Although nutrient enrichment is known to drive excessive algal and microbial growth, which can directly and indirectly change the ecological community composition, the resulting changes in food web emergent properties are poorly understood. We used ecological network analysis (ENA) to examine the emergent properties of 12 riverine food webs across a nutrient enrichment gradient in the Manawatu, New Zealand. We also derive Keystone Sensitivity Indices to explore whether nutrients change the trophic importance of species in a way that alters the resilience of the communities to further nutrient enrichment or floods. Nutrient enrichment resulted in communities composed of energy inefficient species with high community (excluding microbes) respiration. Community respiration was several times greater in enriched communities, and this may drive hypoxic conditions even without concomitant changes in microbial respiration. Enriched communities exhibited weaker trophic cascades, which may yield greater robustness to energy flow loss. Interestingly, enriched communities were also more structurally and functionally affected by species sensitive to flow disturbance making these communities more vulnerable to floods.

## Graphical abstract

The food webs of rivers across a nutrient gradient were examined. Enriched webs are likely to be more stable to random disturbances, but less stable to disturbance from floods or further nutrient enrichment. Enriched webs were also composed of relatively inefficient species that showed substantially greater community respiration.

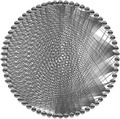

## 1 INTRODUCTION

The eutrophication of rivers, lakes, and groundwater is increasing rapidly, and among the most influential drivers of the global decline in aquatic biodiversity (Dudgeon, 2014; Dudgeon et al., 2006; Vorosmarty et al., 2010). Many regions worldwide now having 10‐ to 15‐fold greater nitrogen flux through their rivers than several decades ago, driven largely by intensive agriculture and wastewater (Glibert, 2017; Howarth, 2008; Le Moal et al., 2019). Dissolved inorganic nitrogen (DIN) and dissolved reactive phosphorus (DRP) are the dominant culprits of eutrophication, both are limiting the growth of both autotrophs (usually algae and macrophytes) and heterotrophs (usually bacteria and invertebrates) (Camargo & Alonso, 2006; Conley et al., 2009; Ferreira et al., 2015; Smith & Schindler, 2009). At very high levels, they can be directly toxic to freshwater organisms (Camargo & Alonso, 2006; Camargo et al., 2005; Hickey & Martin, 2009), but can result in hypoxic conditions if decomposition increases excessively. Nutrients promote the growth of microbes, which can rot dead organic matter, often nutrient‐promoted excessive algae coincide and fuel this, with the microbial decomposition dramatically reducing ambient dissolved oxygen levels and changing pH, which in turn have adverse effects on instream invertebrates and fish (Dodds & Smith, 2016; Ferreira et al., 2015; Hilton et al., 2006; Smith et al., 2006). Furthermore, microbes can mine their environment for nutrients and condition nutrient‐poor detritus, in turn, increasing the palatability for detritus‐consuming invertebrates and relieving nutrient constrained growth (Evans‐White & Halvorson, 2017; Ferreira et al., 2015; Guo et al., 2016; Hessen et al., 2013). Nutrient enrichment typically changes riverine invertebrate communities from being mayfly, stonefly, and caddisfly dominated to those dominated by worms, snails, and midges (Ballantine & Davies‐Colley, 2014; Cullen et al., 2006; Tonkin et al., 2013). Surviving fish species may have poor condition as a result of stress and the dietary changes from the altered macroinvertebrate communities (Baker et al., 2013; Schreck & Tort, 2016; Sealey & Gatlin, 1999; Thera et al., 2020).

While riverine community composition changes with nutrient enrichment (Camargo & Alonso, 2006; Smith & Schindler, 2009), the influence of those changes on food web emergent properties/functioning is not well understood (Boersma et al., 2008; Dodds, 2007; Friberg et al., 2011; Price et al., 2019). Theoretical investigations have hypothesized that nutrient enrichment should yield increases in the relative importance of dietary specialists, increase the amount of energy flowing through the food web, drive longer food chains, alter nutrient stoichiometry, increase population variability, and reduce the amount of cycling (DeAngelis, 2012). Ecological network analysis (ENA) provides a toolbox of mathematical measures to quantify characteristics of weighted food webs, such as cycling and dietary specialists (Fath & Borrett, 2006; Fath & Patten, 1999; Latham Ii, 2006). Despite the potential application of ENA to inform how aquatic food web functioning differs with nutrient enrichment, the few empirical applications of ENA across enriched aquatic systems have yielded inconsistent results (Almunia et al., 1999; Baeta et al., 2011; Mukherjee et al., 2015; Patricio et al., 2006).

A difficult property of ecological communities to quantify is food web stability. Food web stability is the maintenance of food web structure and function over time, and it includes resilience, persistence, equilibrium, resistance, and robustness (Dunne et al., 2005; Mougi & Kondoh, 2016; Rooney & McCann, 2012; Saint‐Béat et al., 2015). Current theory suggests that having many relatively weak indirect flows can stabilize webs by dampening the spread of destructive cascades (Canning & Death, 2017; Mougi & Kondoh, 2016; Rooney & McCann, 2012; Saint‐Béat et al., 2015). When disturbances are species‐specific, secondary extinctions are most likely to occur when species with the highest link weight (both direct and indirect effects) are perturbed (Zhang et al., 2016). Analysis of un‐weighted food webs also suggested species connectivity had high influence on secondary species extinctions (Canning & Death, 2018; Dunne et al., 2002; Montoya et al., 2006); however, connectivity has since been shown to be unimportant in weighted food webs (Zhang et al., 2016). Species with high influence on food web stability are often termed as being keystone (Mills et al., 1993). Therefore, if food webs change in a way that means the most sensitive species to disturbance are also keystone species, then the food web will likely have lower stability to the said disturbance.

Rivers often face multiple disturbances (both natural and anthropogenic), and some taxa may be resilient to one type of disturbance but sensitive to another. If all species within a community can be assigned a score of how keystone they are and how sensitive they are to a given disturbance, then the overall potential sensitivity of the food web to the given disturbance could be estimated by averaging the keystone scores of each species and weighting this by their respective disturbance sensitivity. High weighted averages would indicate a community with keystone species that are also sensitive to the given disturbance, which may mean that community is less stable to the disturbance than a community with a lower weighted‐average.

New Zealand, like many developed countries, has experienced considerable decline in ecological health over the last 25 years—largely from eutrophication in lowland, agriculture‐dominated catchments (Foote et al., 2015; Joy et al., 2019; Julian et al., 2017; Larned et al., 2016). Exacerbations of existing nutrient enrichment and natural floods are two of the most common disturbances facing rivers. Furthermore, for most New Zealand riverine macroinvertebrates there are well‐established scores of their sensitivity to nutrient enrichment and well‐established relationships between body traits and resilience to floods (Scarsbrook & Townsend, 1993; Stark & Maxted, 2007; Townsend et al., 2003). In this study, we assembled weighted food webs for twelve rivers across a nutrient gradient within the Manawatu, New Zealand. First, we used ENA to explore whether overall food web functioning differed across the nutrient gradient. Secondly, we scored the macroinvertebrate assemblages on their sensitivity to further nutrient enrichment and floods, and scored how keystone they are, to produce indices of the community sensitivity (termed here Keystone Sensitivity Indices) to further nutrient enrichment or floods. We then assessed whether these sensitivity indexes changed across the nutrient gradient.

## 2 METHODS

### 2.1 Study sites

Twelve sites in the Manawatu River catchment varying in nutrient enrichment were studied (Table 1). At each site, DIN and DRP concentrations were the average of 7 years (1999–2006) of monthly samples, collected as a part of state of environment monitoring, and processed in accordance with NZS/ISO/IEC 17025:1999 protocols (Death & Death, 2006). A 7‐year monitoring period was chosen as single grab samples are highly variable and the observed ecological communities would have developed over multiple years; longer than 7 years may run the risk of detecting change rather than state (ANZG, 2018).

**Table 1:** The average dissolved inorganic nitrogen concentration (DIN, mg/L), dissolved reactive phosphorus (DRP, mg/L), species richness (N), connectance (C), total system throughflow (TST, J/m^2^/yr), and locations (WGS 84) of twelve river food webs sampled in 2007 in the Manawatu, NZ

Site	Latitude	Longitude	DIN	DRP	N	C	TST
Mangatainoka River @ Putara Road	40°40″49.9′S	175°32″50.8′E	0.02	0.003	33	0.11	8,749,303
Pohangina River @ Piripiri Road	40°03″04.9′S	175°56″11.5′E	0.05	0.005	62	0.09	16,377,036
Pohangina River @ Raumai Reserve	40°12″26.3′S	175°47″21.5′E	0.08	0.012	53	0.12	6,629,034
Tokomaru River @ Horseshoe Bend Reserve	40°29″14.3′S	175°31″33.4′E	0.08	0.005	67	0.12	19,844,310
Raparapawai Stream @ Jacksons Road	40°19″25.2′S	175°59″45.8′E	0.15	0.030	62	0.11	20,767,724
Oroua River @ Nelson Street	40°13″50.0′S	175°34″55.1′E	0.24	0.010	38	0.12	11,872,967
Manawatu River @ SH2	40°23″60.7′S	175°53″17.0′E	0.40	0.009	64	0.12	34,246,823
Mangatera Stream @ SH2	40°14″27.1′S	176°05″55.6′E	0.44	0.141	62	0.10	54,782,015
Oroua River @ Awahuri Road	40°16″32.4′S	175°31″17.6′E	0.53	0.101	37	0.09	34,819,550
Mangatera Stream @ Timber Bay	40°14″27.1′S	176°05″55.6′E	0.81	0.141	66	0.11	40,277,332
Manawatu River @ Hopelands Road	40°21″35.3′S	175°57″42.1′E	0.90	0.024	64	0.11	22,250,529
Mangapapa Stream @ Troup Road	40°20″31.0′S	175°50″52.3′E	0.90	0.024	67	0.12	40,237,153

Note

Nutrient concentrations were based on 84 sampling occasions, sampled monthly between 1999 and 2006 inclusive

### 2.2 Food web construction

Food webs are ecological networks of who eats whom within an ecological community. At all twelve sites, the food webs were assembled to represent plausible Summer 2006/2007 configurations with currency being joules of energy. The main groups included in the food web models were fish, macroinvertebrates, periphyton, and detritus; all living compartments were resolved to the lowest taxonomic keys using the relevant keys available (see below for more details). Microbes were excluded because there was a lack of suitable data available, and the large energy flow through a single microbial compartment may result in uneven resolution and obscure network patterns (Abarca‐Arenas & Ulanowicz, 2002; Baird et al., 2009; Fath et al., 2013). Omitting microbes will, however, underestimate total system throughflow and total system cycling, so readers should keep this in mind when comparing against webs assembled with microbes. The food webs were assembled in WAND (Allesina & Bondavalli, 2004) format. For consumers, rates of consumption were assumed equal to productivity + respiration + unassimilated food. Where consumption exceeded that estimated to be produced locally, it was assumed that invertebrate and detritus drift into the local community accounted for the shortfall to ensure the webs were energetically balanced. Where species were predicted to grow faster than they are consumed, then outputs set equal to any net gains in a species biomass (i.e., drift downstream, death, and microbial processing).

To depict the quality of model parameters, following the data quality rating framework proposed by Costanza et al. (1992), qualitative confidence levels were assigned to model parameters for each group (Table 2). High indicates direct measurement of field data, medium indicates indirect measurements, calculated data, or handbook estimates, while low indicates educated guesses, very indirect approximations, and “rule of thumb” estimates (Costanza et al., 1992).

**Table 2:** The confidence ratings of model parameters following Costanza et al. (1992)

Group	Individual densities	Biomass conversion	Productivity	Respiration	Assimilation efficiency	Diet	Boundary flows
Periphyton	High	Medium	Medium	Medium	N/A	N/A	Low
Invertebrates	High	Medium	Medium	Medium	Medium	Medium	Low
Fish	High	Medium	Medium	Medium	Medium	Medium	Low
Detritus	Low	Low	N/A	N/A	N/A	N/A	Low

#### 2.2.1 Basal compartments

Periphyton biomass was approximated from the average chlorophyll a (mg/m^2^) measured between January and March 2007. At each site, chlorophyll a density was estimated from five unglazed tiles (0.25 m × 0.25 m) and from five randomly collected stones. Stone surface area was estimated following Graham et al. (1988) with periphyton assumed to be covering half of the total surface area. Pigments were collected separately for each tile or stone in known volumes of 90% acetone at 5°C in the dark for 24 hr. Pigment densities were converted from absorbances determined using a Varian Cary 50 Conc UV‐visible Spectrophotometer, following Steinman et al. (1996). Algal species composition was also determined from stone scrapings following Steinman et al. (1996). The approximate biomass of each species was determined by rationing the total periphyton biomass (from chlorophyll a–biomass relationships) by relative species abundance (Banse, 1977; Brey et al., 2010; Keenan & Morar, 2015). Periphyton annual production/biomass (P/B) was estimated to be 35 by extrapolating the hourly rate determined in Elwood and Nelson (1972). The annual respiration/biomass (R/B) rate was approximated to be 10.95 following an approximate daily R/B of 0.03 (0.03 over 365 days is 10.95) calculated by McIntire and Phinney (1965).

#### 2.2.2 Invertebrate compartments

At each site, macroinvertebrates were sampled using five randomly positioned Surber samplers (0.1‐m^2^ and 250‐µm mesh) between January and March 2007. Invertebrates within the 0.1‐m^2^ quadrat were scrubbed off all cobbles and fine sediment stirred down to a depth of approximately 10 cm for 1 min. Samples were preserved in 10% formalin, sorted, and enumerated to the lowest practicable taxonomic level (Winterbourn et al., 1989).

Mean individual lengths and biomasses for each species were determined from the literature and length‐biomass regressions (Moore, 1998; Stoffels et al., 2003; Towers et al., 1994; Winterbourn et al., 1989). Invertebrate biomass (dry weight) was converted to Joules following Brey et al. (2010). The annual production and respiration were estimated using empirical models for aquatic invertebrates with a typical annual median temperature of 14°C (Brey, 2010, 2012; Death & Death, 2006). The estimated production rates were similar to those derived for the same or similar taxa throughout other parts of New Zealand (Collier et al., 2004; Hopkins, 1971; Huryn, 1996, 1998; Tank & Winterbourn, 1996). Rates of consumption were assumed equal to productivity + respiration + unassimilated food.

The presence of dietary links between species was estimated from their functional feeding group, and predator diets were established from the literature (Collier et al., 2004; Cowley, 1978; Devonport & Winterbourn, 1976; Hollows et al., 2002; Jaarsma et al., 1998; McFarlane, 1976; Polegatto & Froehlich, 2003; Rounick & Hicks, 1985; Thompson & Townsend, 2004; Towns & Peters, 1996; Winterbourn, 1978, 1982, 1996, 2000; Winterbourn et al., 1984, 1989). Dietary proportions of each resource were assumed proportional to the productivity of prey/basal taxa unless literature indicated there was strong dietary preference. The assimilation efficiency (assimilation/ingestion) of dietary components was the same as those used in Benke et al. (2001).

#### 2.2.3 Fish compartments

Fish density and approximate lengths were determined from electric fishing surveys collected during the 2006/2007 Summer period (same period as the macroinvertebrate surveys) and recorded in the New Zealand Freshwater Fish Database (Richardson, 1989).

Fish biomass was estimated from approximate lengths following the length–mass equations in Jellyman et al. (2013). Fish productivity rates were assumed to be the same as those determined in neighboring streams (Hopkins, 1971). Clarke and Johnston (1999) was used to estimate respiration rates assuming typical median temperature is 14°C (Death & Death, 2006).

Fish diet was determined from previous dietary studies, and flows were proportional to the abundance of prey biomass unless literature indicated a high dietary preference (Cadwallader, 1975; Glova & Sagar, 1989, 1991; Jellyman, 1996; Kusabs & Swales, 1991; Main & Winterbourn, 1987; Montori et al., 2006; Sagar & Glova, 1995).

#### 2.2.4 Detrital compartments

Two detrital compartments were used to represent fine particulate organic matter (FPOM) and course particulate organic matter (CPOM). All organisms that died from causes other than consumption entered the CPOM pool. Detritus compartment storages were all set to a nominal figure of 100 J/m^2^ and steady state maintained by assuming imports (i.e., upstream vegetation and detritus flowing into the reach) equaled the outputs from invertebrate consumption. The assumption of steady state is likely unrealistic; however, the network analyses used in this study rely on networks being balanced at equilibrium (i.e., at steady state) and should be interpreted as snapshot assessments. The nominal detritus storage value is relatively small and will have a consistent, but small, effect on the total system storage that readers should be aware of. The nominal detritus storage value will have no effect on flow‐based metrics.

### 2.3 Food web analysis

**Figure 1:**
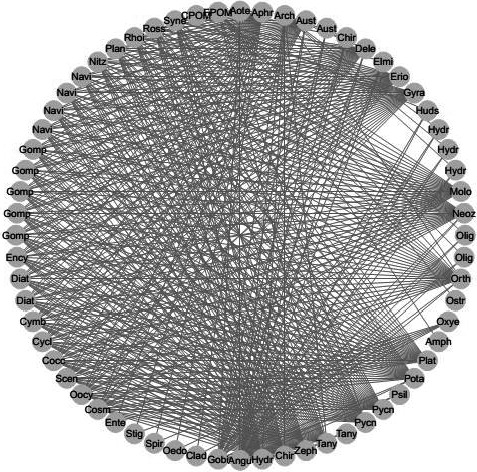
The linkages within food web assessed at the Manawatu River at Hopelands Road in Summer 2007. Letters are the first four characters of each taxon name

For each web (e.g., Figure 1.), network metrics were calculated using the functions from the enaR package (Borrett & Lau, 2014) and implemented within R 3.3.1 (R Core Team, 2016). Initially, any remaining small imbalances were balanced using the AVG2 method described by Allesina and Bondavalli (2003) prior to calculating the following metrics: total system throughflow, sum of loss to respiration, sum of loss to exports, total system storage, network aggradation, indirect flow intensity, homogenization (inputs), relative ascendency, flow‐based network synergism, and the flow‐based network mutualism (Borrett et al., 2006; Fath, 2007; Fath & Patten, 1999; Finn, 1976; Ulanowicz, 1997a). The AVG2 balancing algorithm was used to balance networks to steady state, an assumption required for computing the network metrics, as this methods typically generate the least distortion of network traits (Allesina & Bondavalli, 2003). Metric interpretations are included in Table 3.

**Table 3:** The regression statistics of ten network metrics, and their definition, across twelve rivers (Manawatu, New Zealand) along a dissolved inorganic nitrogen (DIN) gradient

Network metric	Abbreviation	Metric interpretation	Relationship	*R* ^2^	*F*‐statistic	*p*‐value
Total system throughflow	TST	Sum of energy flows entering or exiting all taxa. It is an indicator of the size of the food web	Log–log	0.58	13.50	.004
Respiration flows	Resp.	Sum of all energy lost from respiration	Log–log	0.54	11.90	.006
Export flows	Exports.	Sum of all energy leaving the system, this could be via death (transfer to microbes), emigration, or drift from floods	Log–log	0.05	0.48	.50
Network aggradation	Net. Agg.	The average food chain length based on weighted energy flows	Linear	0.03	0.03	.58
Indirect flow intensity	IFI	Proportion of all energy flows that has arisen from indirect flows. Indirect flows are energy that has been transferred from one species to another via an intermediate species, such as through trophic cascades	Log‐linear	0.53	10.02	.01
Homogenization (input, flow)	Hmg (input)	A maximally homogeneous network is one in which species receive an equal amount of energy from each of the other species in the community and distributes an equal amount to all the other species (either directly or indirectly)	Linear	0.05	0.51	.49
Relative ascendency	Rel. Asc.	Indicates both the size and the complexity of a web. A low relative ascendency indicates a web with low energy flow and/or many generalists. A high relative ascendency indicates a web with high energy flow and/or many specialists	Quadratic	0.64	6.97	.02
Total system storage	TSS	Sum of energy stored in all taxa. It is an indicator of the size of the community	Linear	0.05	0.52	.49
Mutualism (flow)	Mut. (flow)	Measure of the obligatory positive feedback flows	Linear	0.11	1.21	.30
Synergism (flow)	Syn. (flow)	Measure of the nonobligatory positive feedback flows	Linear	0.73	24.6	<.001

Note

DF = 10.

Regression analysis using R 3.3.1 (R Core Team, 2016) was used to test the relationship between each network metric calculated (the network metrics in Table 3 all comprised as independent variables) and the respective DIN concentration (dependent variable) for all twelve webs. Given that DIN concentration was significantly correlated with DRP (log–log‐transformed, *r* ^2^ = 0.56, *F*_1,10_ = 12.51, *p* = .005), we only regressed metrics with DIN. Here, we use DIN as the indicator of nutrient enrichment and do not necessarily consider it the only limiting factor.

### 2.4 Keystone macroinvertebrate sensitivity

Two common disturbances in New Zealand rivers are increasing nutrient enrichment and floods. If keystone food web invertebrates are sensitive to high nutrients, then the web will be sensitive to increasing nutrient enrichment. Likewise, if keystone species have only one terrestrial life stage, are crawlers, have cylindrical body forms, exposed gills, and low dissemination potential, then the communities may be more sensitive to flow disturbance than those with keystone taxa that have multiple terrestrial life stages, are burrowers, have streamlined body forms, plastron respiration, and high dissemination potential (McIntosh et al., 1994; Scarsbrook & Townsend, 1993; Townsend et al., 2003).

To investigate whether nutrient enrichment changed the web‐wide influence of disturbance‐sensitive macroinvertebrates, we calculated five keystone sensitivity indexes (KSI) for each web. Each of the five KSIs was based on five species trait categories known to be influenced by enrichment or flow disturbance, being: body form, locomotion, means of respiration, dispersal potential, and organic enrichment sensitivity. The KSIs for each web and each trait were calculated following Equation 1.

(1) $$\mathrm{KSI}=\frac{\sum_{m}\mathrm{Sensitivity}\mathrm{score}\times \mathrm{Average}\mathrm{environ}\mathrm{centrality}}{\sum_{m}\mathrm{Average}\mathrm{environ}\mathrm{centrality}}$$

where *m* is each of the macroinvertebrate species within a given food web.

The average environ centrality (AEC) is an indicator of the web‐wide influence a species has and measures a species web‐wide direct and indirect effect (Fann & Borrett, 2012) and was calculated for each species in each web using enaR (Borrett & Lau, 2014). The AEC values represent the relative contribution of a species, as a proportion, to the total energy‐matter exchange within a system, with all species summing to one—permitting comparison between webs.

Species traits were sourced from the Freshwater Biodata Information System (National Institute for Water & Atmospheric Research, 2004), and trait‐specific sensitivity scores were assigned as follows (higher values being more sensitive):

1. **Body form**: Cylindrical (3), flattened (2), and streamlined (1).
2. **Locomotion**: Crawler (3), swimmer (2), and burrower/sessile (1).
3. **Respiration/gas exchange mechanism**: Gills/aerial (3), tegument (2), and plastron (1).
4. **Dispersal ability**: Low (3), moderate (2), and high (1)
5. **Organic enrichment sensitivity**: Individual species sensitivity scores adopted from the MCI (Clapcott et al., 2017; Stark & Maxted, 2007).

Using regression analysis in R 3.3.1 (R Core Team, 2016), the relationships between each of five trait‐specific KSIs (each as a dependent variable) and DIN concentration (independent variable) were examined (Table 3). Linear regressions were used with Box‐Cox transformations (Osborne, 2010) applied to DIN (where necessary) using the MASS package (Ripley et al., 2013).

## 3 RESULTS

Across all twelve food webs, the mean dissolved inorganic nitrogen (DIN) concentration ranged from 0.05 to 0.90 mg/L, taxonomic richness ranged from 33 to 67, and total system throughflow from 6,629,034 to 54,782,015 J/m^2^/year (Table 1).

**Figure 2:**
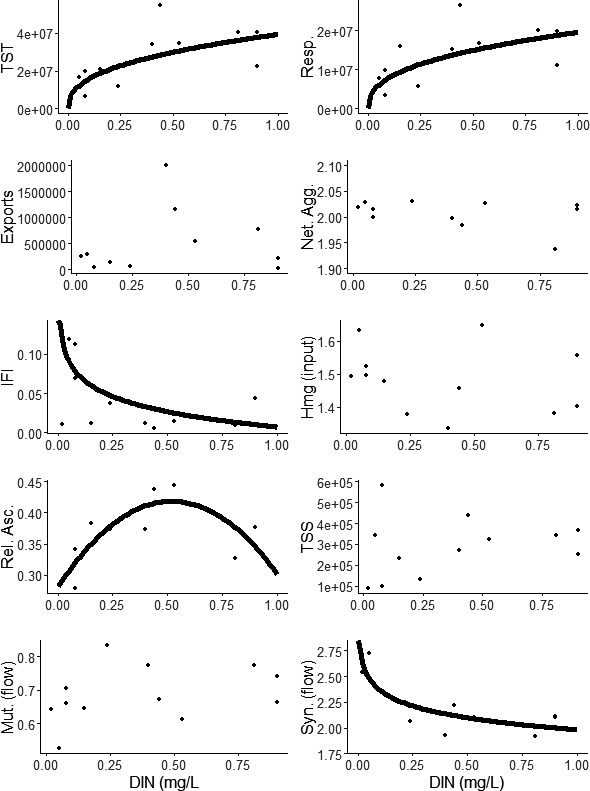
Ten network metrics from riverine food webs across a dissolved organic nitrogen (DIN, mg/L) gradient throughout the Manawatu, NZ. Lines represent statistically significant regressions (*p* < .05, Table 3)

As DIN concentration increased, total system throughflow, total respiration, and the indirect flow intensity all increased up to a point, after which values remained similar despite increasing DIN. Synergism declined slightly with increasing DIN also until a threshold was met, after which values remained similar with increasing DIN. There was no relationship between DIN and exports, homogenization, total system storage, and mutualism. Relative ascendency increased with increasing DIN until a threshold, after which relative ascendency reduced with increasing DIN (Figure 2, Table 3).

**Figure 3:**
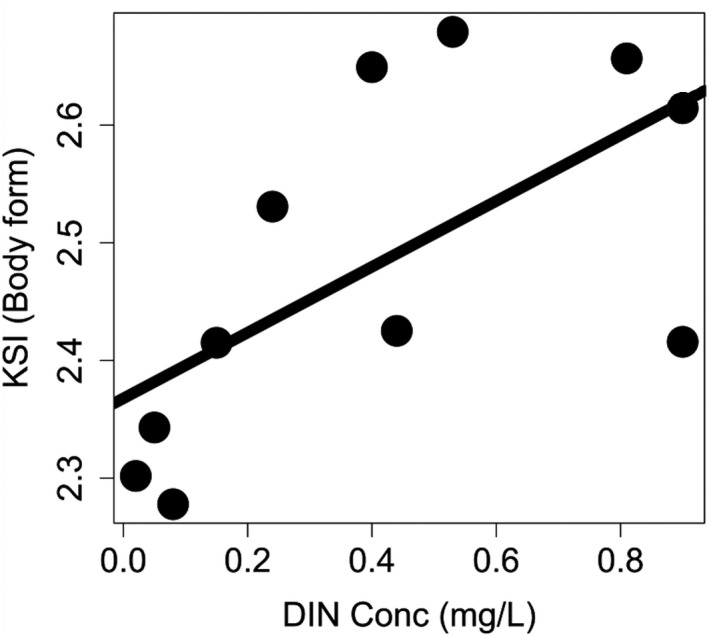
The Keystone Sensitivity Index (body form) across riverine food webs along a dissolved organic nitrogen (DIN, mg/L) gradient throughout the Manawatu, NZ. Linear regression line is statistically significant (*F*_1,10_ = 6.02, *p* = .04)

Of the disturbance sensitivity indices, only the body form sensitivity index increased with DIN concentration, while the remainder were not correlated with DIN (Figure 3, Table 4).

**Table 4:** The regression statistics for five Keystone Sensitivity Indices (KSI) across twelve rivers (Manawatu, NZ) along a dissolved inorganic nitrogen (DIN) gradient

Keystone Sensitivity Index trait	*R* ^2^	*F*‐statistic	*p*‐value (>|*F*|)
Body form	0.40	6.0	.04
Locomotion	0.003	0.03	.86
Respiratory mechanism	0.001	0.01	.92
Dispersal potential	0.03	0.35	.57
Organic enrichment sensitivity	0.001	0.01	.92

Note

DF = 10.

## 4 DISCUSSION

Total energy flow increased (measured using TST) with increasing nutrient enrichment until a DIN concentration of approximately 0.5 mg/L after which total energy flow remained constant. The initial increase in throughflow is consistent with bottom‐up control theory whereby an increase in algal and microbial productivity (in this case from greater nutrient enrichment) results in increasing productivity of consumers (Bumpers et al., 2017; Cross et al., 2007; Ferreira et al., 2015). The total food web biomass was unaffected by nutrient enrichment, rather the per‐capita throughflow of energy increased instead. Thereby indicating that either: (a) production increased and organisms moved across the system boundary (organisms drifted or swam out of the area or died) thereby maintaining total system biomass and increasing throughflow; or (b) the webs are composed of more energetically demanding/more inefficient species that require greater energy consumption resulting in increased throughflow. Interestingly, while exports (nonrespiratory boundary flows out of the system) remained unchanged, total community respiration increased markedly with increasing nutrient enrichment. This supports the latter hypothesis that the communities became composed of energetically inefficient species with increasing enrichment. Hypoxia is often associated with the microbial decomposition of algal blooms and detritus (Hilton et al., 2006; de Jonge et al., 2002; Smith et al., 2006); however, our results show that at DIN concentrations greater than approximately 0.35 mg/L that respiratory energy demand, and consequently oxygen demand, for the entire community excluding microbes can be three to four times greater than in oligotrophic conditions. The change in invertebrate community composition could therefore also be having a considerable influence on the frequency and intensity of hypoxic conditions without the need to invoke any microbial processes.

Nutrient enrichment may have resulted in an increase in the composition of inefficient species by increasing the frequency and/or intensity of both hypoxic and substrate movement disturbances. Highly disturbed communities tend to be composed of smaller bodied species as they are typically fast growing and can recover more easily (Dinh & Death, 2018; Dolédec et al., 2006; Townsend et al., 2003). While a small‐bodied invertebrate on its own will likely have low respiration demand, when biomass differences are accounted for smaller bodied invertebrates tend to have greater respiration rates than larger invertebrates (Brey, 2010; Gillooly et al., 2001; Robinson et al., 1983); thus, making communities with smaller invertebrates less energy efficient than larger invertebrates assuming total biomass is the same. Eutrophic streams in general have more prolific periphyton growth. Prolific periphyton can disturb communities by increasing the frequency and intensity of hypoxic events (Dodds & Smith, 2016; Glibert, 2017; Le Moal et al., 2019). Also, if periphyton dominates the base of the food web, then substrate movement (which scours periphyton from rocks) will also be disturbing for the invertebrates that rely on periphyton (Canning et al., 2017, 2019; Lake, 2000; Marks et al., 2000). Therefore, if hypoxic events and periphyton scouring disturb the community, then the increased frequency and/or intensity of stream disturbances may drive more enriched communities to be composed of small‐bodied, energetically inefficient species (Hilton et al., 2006; Townsend & Hildrew, 1994).

Similarly to total energy flow, the confinement of energy flows (measured by relative ascendency) also increased with enrichment until an approximate DIN concentration of 0.50–0.55 mg/L, after which energy flow confinement relaxed slightly. Mageau et al. (1998) suggested that relative ascendency would increase with nutrient enrichment; however, they did not account for the potential effects of a dominant competitor collapsing which would revert the network to a state with relatively unconstrained energy flows (the large, specialized flows of dominant competitor are lost). In river systems, nutrient enrichment may increase algal growth and drive more energy through the algal consumers, thereby increasing relative ascendency (Almunia et al., 1999; Ulanowicz, 1986, 1997a; Zorach & Ulanowicz, 2003). Relative ascendency may also increase if algal composition changes in way where some species are preferable for invertebrates than others, and the food web becomes skewed toward preferable or more rewarding species (Guo et al., 2016; Guo, Kainz, Valdez, et al., 2016). As explained above, prolific periphyton growth may increase disturbance frequency and/or intensity (Hilton et al., 2006). Disturbance often returns communities to earlier successional stages that have more uniform energy flow distributions (i.e., relative ascendency drops) (Genoni & Pahl‐Wostl, 1991; Mageau et al., 1998; Ulanowicz, 1997b). It is plausible that when DIN concentrations exceed 0.55 mg/L that hypoxic events occur and disturb the system sufficiently to drop relative ascendency. Ulanowicz (1997b) found that most weighted food webs have very similar relative ascendency values, which has been suggested to represent a common trade‐off between adaptable yet inefficient communities and efficient yet brittle communities—termed the window of vitality (Canning & Death, 2018; Ulanowicz, 2009; Ulanowicz et al., 2009). Interestingly, all the webs we assessed were within the window of vitality, with the webs of highest relative ascendency occurring approximately at the upper boundary of the established window.

The indirect flow intensity (measures the dominance of trophic cascades) decreased sharply with increasing nutrient enrichment until an inflection at DIN concentrations of approximately 0.3–0.4 mg/L, after which indirect flows remained consistently at very low levels of total system flow. Weak indirect effects have been shown to increase food web robustness as they dampen the spread of disruptive cascades induced by disturbance. On the contrary, strong indirect effects have been associated with greater biocomplexity by supporting species coexistence through increased heterogeneity in space and time. Therefore, increasing enrichment may stabilize webs yet reduce their biocomplexity by reducing the proportion of indirect flows.

Synergistic effects, being nonobligatory positive feedback relationships (Fath & Patten, 1998), also reduced slightly with increasing enrichment between DIN concentrations of approximately 0.10–0.20 mg/L, after which synergism remained unaffected by increasing DIN. Despite differences in synergism, mutualistic effects, being obligatory positive feedback relationships (Fath, 2007), were unaffected by increasing DIN. Synergistic energy flows allow for the simultaneous growth of multiple species. Since species in synergistic relationships are not reliant on each other for survival but complimented, then if one species in a synergistic loop is perturbed then the others will support the recovery of the perturbed species, thereby supporting food web resilience (the ability of a food web to recover following disturbance) (Okuyama & Holland, 2008; Saint‐Béat et al., 2015). The reduction in synergism with nutrient enrichment may, therefore, reduce resilience.

While our analysis used nutrient samples that averaged over a 7‐year period, the biological communities were a single snapshot at the end of that period. This decision may have resulted in a mismatch between measured nutrient concentrations and measured biological communities, particularly for periphyton biomass, which can also be variable. However, we consider that long‐term and regular nutrient samples are required to establish the nutrient enrichment status of a stream as singular grab samples are highly variable. Large nutrient variability can arise from nutrient discharge practices, rainfall patterns, and the biological exchange of nutrients. Biological communities develop over long periods and are exposed to a wide variety of nutrient concentrations over that period. If our analysis was to only use a single nutrient grab sample at the time of biological community collection, then the assessed nutrient enrichment status may not be representative of that exposed to the biological community over the long term. Therefore, the latter approach may have resulted in different relationships to that observed in this analysis.

If nutrient enrichment shifts disturbance‐sensitive species from positions of low influence to positions of high influence, then their loss during the next disturbance would likely be more pervasive than when they were less influential (Zhao et al., 2016). We found that as nutrient enrichment increased, the community structure changed in a way that meant species whose body forms were sensitive to flood disturbances became more influential (greater environ centrality; Figure 3). Therefore, in our webs, floods are more likely to cause disruptive cascades and destabilize webs that are highly enriched as species with body forms that are more easily disturbed by flood disturbances are shifted to more influential positions.

In conclusion, as nutrient enrichment increased, the communities became energy inefficient as community (excluding microbes) respiration increased. Increased community oxygen demand may drive or exacerbate hypoxic events. Furthermore, the strength of trophic cascades reduced with increasing enrichment, this may reduce the effects of disruptive cascades. Interestingly, enriched communities were also structured in a way that resulted in flood‐sensitive species (based on body form) being more influential than in pristine communities; therefore, the communities may be more sensitive to collapse from flow disturbance.

## CONFLICTS OF INTEREST

None declared.

## AUTHOR CONTRIBUTIONS

**Adam D. Canning:** Conceptualization (lead); data curation (supporting); formal analysis (lead); funding acquisition (equal); investigation (lead); methodology (lead); project administration (lead). **Russell G. Death:** Conceptualization (equal); data curation (lead); methodology (equal); supervision (lead); writing–original draft (supporting); writing–review and editing (equal).

## Data Availability

Compiled food webs available at: https://doi.org/10.5061/dryad.fqz612jrh.
